# Storage and allogeneic transplantation of peripheral nerve using a green tea polyphenol solution in a canine model

**DOI:** 10.1186/1749-7221-5-17

**Published:** 2010-11-28

**Authors:** Ken Nakayama, Ryosuke Kakinoki, Ryosuke Ikeguchi, Tomoyuki Yamakawa, Soichi Ohta, Satoshi Fujita, Takashi Noguchi, Scott FM Duncan, Suong-Hyu Hyon, Takashi Nakamura

**Affiliations:** 1 Department of Orthopedic Surgery, Shizuoka General Hospital, Shizuoka, Japan; 2 Department of Orthopedic Surgery, Graduate School of Medicine, Kyoto University, Kyoto, Japan; 3 Department of Rehabilitation Medicine, Kyoto University Hospital, Kyoto, Japan; 4 Department of Orthopedic Surgery, Kobe Central Municipal Hospital, Kobe, Japan; 5 Department of Orthopedic Surgery, Tango Central Hospital, Tango, Japan; 6 Department of Orthopedic Surgery, Mayo Health System, Owatonna, USA; 7 Institute for Frontier Medical Science, Kyoto University, Kyoto, Japan

## Abstract

**Background:**

In our previous study, allogeneic-transplanted peripheral nerve segments preserved for one month in a polyphenol solution at 4°C could regenerate nerves in rodents demonstrated the same extent of nerve regeneration as isogeneic fresh nerve grafts. The present study investigated whether the same results could be obtained in a canine model.

**Methods:**

A sciatic nerve was harvested from a male beagle dog, divided into fascicules of < 1.5 mm diameter, and stored in a polyphenol solution (1 mg/ml) for one month at 4°C. The nerve fascicles were transplanted into 10 female beagle dogs to bridge 3-cm right ulnar nerve gaps. In the left ulnar nerve in each dog, a 3-cm nerve segment was harvested, turned in the opposite direction, and sutured in situ. Starting one day before transplantation, the immunosuppressant FK506 was administered subcutaneously at doses of 0.1 mg/kg daily in four dogs (PA0.1 group), 0.05 mg/kg daily in four dogs (PA0.05 group), or 0.05 mg/kg every other day in two dogs (PA0.025 group). Twelve weeks after surgery, electrophysiological and morphological studies were performed to assess the regeneration of the right and left ulnar nerves. The data for the right ulnar nerve were expressed as percentages relative to the left ulnar nerve. Polymerase chain reaction (PCR) was used to identify the sex-determining region of the Y-chromosome (*Sry*) and β-actin to investigate whether cells of donor origin remained in the allogeneic nerve segments. FK506 concentration was measured in blood samples taken before the animals were killed.

**Results:**

The total myelinated axon numbers and amplitudes of the muscle action potentials correlated significantly with the blood FK506 concentration. Few axons were observed in the allogeneic-transplanted nerve segments in the PA0.025 group. PCR showed clear *Sry*-specific bands in specimens from the PA0.1 and PA0.05 groups but not from the PA0.025 group.

**Conclusions:**

Successful nerve regeneration was observed in the polyphenol-treated nerve allografts when transplanted in association with a therapeutic dose of FK506. The data indicate that polyphenols can protect nerve tissue from ischemic damage for one month; however, the effects of immune suppression seem insufficient to permit allogeneic transplantation of peripheral nerves in a canine model.

## Background

Autogenous nerve grafting is a widely accepted method for treating peripheral nerve injuries with nerve deficits. However, the sources of donor nerves are limited, and donor site morbidity is inevitable. The shortage of nerve sources for transplantation is a serious problem in autogenous nerve grafting. Nerve allografts performed in association with the administration of an immunosuppressant provide an alternative to autogenous nerve grafts [1,2]. However, nonspecific immunosuppressive treatments are often followed by opportunistic infection of nonpathological viruses or neoplasm formation [3-5]. It is controversial whether immunosuppressants should be administered with nerve allografts when repairing peripheral nerve injuries with nerve deficits because such injuries are not usually life threatening.

Green tea polyphenols are intriguing because they protect tissues from ischemia, have antineoplastic and anti-inflammatory effects, and suppress immune responses [6-9]. In our previous studies, peripheral nerve allografts preserved for one month in a green tea polyphenol solution were able to regenerate nerves in a manner similar to that of fresh nerve autografts in a rodent model, suggesting that peripheral nerve segments treated with green tea polyphenols might provide an alternative to autogenous nerve grafts. The observation that the nerve segments could be preserved for one month in the green tea polyphenol solution suggested that treatment with polyphenols could change allogeneic nerve transplantation from an emergency operation to a scheduled operation. However, in our semiquantitative PCR study, only 14% of cells survived in the polyphenol-treated allografts, whereas about 62% of cells survived in the fresh nerve isografts [10]. These results suggested that nerve regeneration from the polyphenol-treated nerve allografts would be inferior to that from fresh nerve isografts when applied to long nerve gaps or in highly developed animals such as dogs, primates, and human beings. We hypothesized that the combination of immunosuppressants administered with polyphenol treatment would lead to successful nerve regeneration with nerve allografts.

In the present study, we performed 3-cm-long allogeneic nerve grafts on the right ulnar nerve and 3-cm-long isogeneic nerve grafts on the left ulnar nerve in a canine model given one of three doses of an immunosuppressant (FK506) [11-13]. We examined the relationship between nerve regeneration in the nerve allografts and blood FK506 level to investigate whether polyphenol treatment can reduce the dosage of the immunosuppressant needed to obtain successful nerve regeneration in the allogeneic nerve transplant.

## Methods

### Polyphenols

A polyphenol mix extracted from green tea was purchased from PFI, Inc. (Kyoto, Japan). It comprised mainly (-)-epigallo-catechin-3-*O*-gallate (28%), (-)-gallocatechin-3-*O*-gallate (11.6%), (-)-epicatechin-3-*O*-gallate (4.6%), (-)-epigallocatechin (15.0%), (+)-gallocatechin (14.8%), (-)-epicatechin (7.0%), and (+)-catechin (9.5%), and its purity exceeded 90%.

### Animals

One male beagle dog (18 kg) and 10 female beagle dogs (15 kg) were used in this study. The male animal was purchased from a different breeder from the one supplying the female animals. Each animal was acclimatized before the surgical procedures, housed in a separate cage, and given standard dog food and water three times a day. All experiments were performed in accordance with the guidelines of the Animal Research Committee, Graduate School of Medicine, Kyoto University, Japan. The female animals were divided into three groups and received subcutaneous injections of one of three doses of FK506: 0.1 mg/kg every day to four animals (PA0.1 group), 0.05 mg/kg every day to four animals (PA0.05 group), and 0.05 mg/kg every other day to two animals (PA0.025 group).

### Surgery

#### Anesthesia

Animals were sedated with 0.2 mg/kg acepromazine given subcutaneously. After intubation, general anesthesia was induced by inhalation with 5% isoflurane. After confirming that the animals were nonresponsive to stimulation of the eyelashes, surgery started.

#### Harvesting nerve allografts and preservation

The male animal was placed in the prone position, and the sciatic nerve was harvested. The sciatic nerve was exposed from the sciatic notch to the popliteal fossa in the interval between the hamstring muscles to obtain a 10-cm-long nerve segment in the midthigh level. The nerve segment was separated into fascicles under an operating microscope, and the fascicles were harvested. The diameter of the fascicles was < 1.5 mm, and the length was > 5 cm [10]. The fascicles were immersed at 4°C in Dulbecco's modified Eagle's medium (DMEM) containing the polyphenols (1 mg/ml) for one week and then transferred to DMEM solution alone and immersed in DMEM for three more weeks.

#### Transplantation of the preserved nerve segments

**Figure 1:**
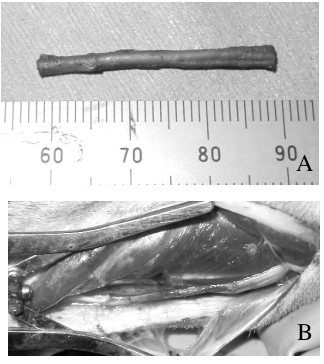
**Nerve transplantation**. A nerve allograft comprising two fascicles of the donor sciatic nerve. (B) An intraoperative photograph of allogeneic nerve transplantation. Two fascicles of the donor sciatic nerve were interposed between the proximal and distal stumps of the ulnar nerve in the middle of the right lower forearm.

Recipient female animals were anesthetized in the supine position as described above, and a skin incision was made on the right lower forelimb. The ulnar nerve was exposed by retracting the ulnar carpal flexor muscle medially and the digital flexors laterally. A 2.5-cm-long ulnar nerve segment was removed at the midportion of the lower forelimb. The sciatic nerve fascicles harvested from the male animal and preserved for one month were taken from the DMEM solution. Because the diameter of the ulnar nerve was almost 2 mm in the middle of the lower forelimb, two fascicles--a thick fascicle of 1.2-1.5-mm diameter and a thin fascicle of 0.5-0.8-mm diameter--were transplanted as the nerve graft. The length of the nerve grafts was adjusted to 3 cm just before transplantation. Each 3-cm-long nerve segment comprising the two fascicles was interposed between the proximal and distal nerve stumps of the ulnar nerve using 10-0 epineural sutures in the right forelimb (the model for allogeneic nerve transplantation) (Figure 1).

In the left forelimb, the ulnar nerve was exposed in the same way as in the right forelimb. A 3-cm-long nerve segment was removed in the middle of the lower forelimb. The nerve segment was turned in the opposite direction and sutured in situ (the model for isogeneic nerve transplantation).

In all animals, FK506 administration was started one day before the transplantation.

### Electrophysiological study

Twelve weeks after nerve transplantation, all recipient animals were anesthetized as described above. The right ulnar nerve was exposed and stimulated 3 cm distal to the elbow joint (S1) and 2 cm proximal to the wrist joint (S2) with a bipolar silver electrode. A pair of plate electrodes was fixed over the prominence of the muscles at the base of the most ulnar digit (hypothenar muscles) to check for the presence of action potentials in the muscles. The motor nerve conduction velocity (MNCV) was calculated for both types of evoked action potentials stimulated at S1 and S2. The amplitude (peak to peak) of action potentials evoked in the hypothenar muscles with the supramaximal electric stimulation was measured. The same procedure was performed on the left forelimb.

The values for the action potential amplitude evoked in the hypothenar muscles and the MNCV for the right ulnar nerve (the allogeneic-transplanted nerve) were expressed as percentages of the values for the left ulnar nerve (the isogeneic-transplanted nerve) and are presented as %action potential amplitude and %MNCV, respectively, for each animal.

### Morphological study

After the electrophysiological study, a 1-cm-long nerve segment was harvested from both ulnar nerves 1 cm distal to the transplanted nerve segments, fixed in 1% glutaraldehyde and 1.44% paraformaldehyde, postfixed with 1% osmic acid, and embedded in epoxy resin. Transverse sections (1 μm thick) were taken from the midportion of the segment. Transverse sections were harvested about 1.5 cm distal to the distal neurorrhaphy of the transplanted nerve segments. The section was stained with 0.5% (w/v) toluidine blue solution and examined by light microscopy. Light microscope images of the specimens were downloaded to a personal computer using Photoshop software (Version 5; Adobe Systems Inc., San Jose, CA). The total number of myelinated axons and mean myelinated axon diameter were calculated on each specimen using Image Pro Plus software (Media Cybernetics, Silver Spring, MD).

Briefly, the entire neural area (a) of each specimen was calculated on an image. Six or seven fields were chosen at random so that the area analyzed would be > 20% of the entire neural area of each specimen. The number of myelinated axons, the neural area, and the shortest diameter of each myelinated axon were calculated for each field. The total number of myelinated axons (b) and neural areas (c) of six or seven fields were calculated. The total number of myelinated axons in each specimen was estimated as b × a/c. The mean myelinated axon diameter (in μm) is expressed as the average value of the shortest diameter of all myelinated axons in the six or seven fields [10,14,15].

The total axon number and mean axon diameter of the right ulnar nerve (the nerve allograft model) are expressed as percentages of those of the left ulnar nerve (the nerve isograft model) and are presented as %total axon number and %mean axon diameter, respectively, for each animal.

### Measurement of blood FK506 concentration and sacrifice of animals

After harvesting the nerve specimens for the morphological study, a 5-ml blood sample was obtained from each animal using percutaneous needle puncture of the heart. A lethal dose of pentobarbital (100 mg/kg/body weight) was then injected into the heart to kill the animals. Blood FK506 concentration was measured in the recipient animals by enzyme-linked immunosorbent assay (ELISA) using an anti-FK506 monoclonal antibody [16].

### Genomic study

Polymerase chain reaction (PCR) was used to investigate the origin of cells in the nerve allografts in the recipient animals using primers specific for the sex-determining region of the canine Y-chromosome (*Sry*) and β-actin genes. After the electrophysiological study, a 1-cm-long nerve segment was taken from the center of each nerve allograft in the right ulnar nerve of all animals. Genomic DNA was extracted from each nerve segment using phenol-chloroform extraction and quantified spectrophotometrically. PCR amplification was performed as described below. The reaction mixture included 0.001 μg of genomic DNA, 0.5 U *Taq *DNA polymerase (AmpliTaq Gold, PerkinElmer Cetus, Shelton, CT), 1 pM of each oligonucleotide primer, 2 μl of a 2.5 mM solution of each dNTP, 2.5 μl of 10× PCR buffer, and 1.5 μl of 1.5 mM MgCl2 in a final volume of 25 μl. The primer sequences used were as follows: canine *Sry *gene, 5'-CTC GCG ATC AAA GGC GCA AGA T-3' and 5'-TTC GGC TTC TGT AAG CAT TTT C-3'; and canine β-actin gene, 5'-TCC TGT GGC ATC CAC GAA ACT-3' and 5'-GAA GCA TTT GCG GTG GAC GAT-3'. PCR was performed in a thermal cycler (PerkinElmer Cetus) for 32 cycles of denaturation (94°C, 45 s), annealing (60°C, 45 s), and extension (72°C, 60 s). The product was analyzed by electrophoresis on a 2% agarose gel followed by ethidium bromide staining [17,18].

### Statistical analysis

Correlation coefficients were calculated between the blood FK506 level and the %action potential amplitudes, %MNCVs, %total axon numbers, and %mean axon diameters using SPSS for Windows (Version 17.0, SPSS Inc., Chicago, IL). A p-value < 0.05 was considered significant.

## Results

### Electrophysiological study

**Figure 2:**
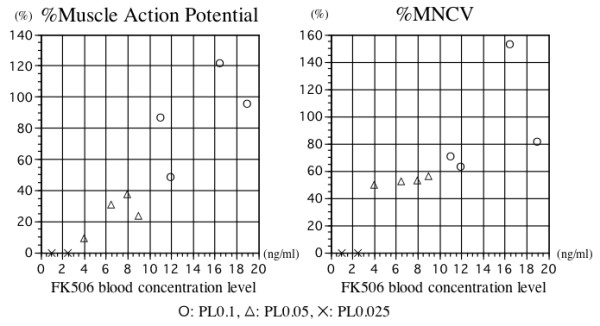
**Electrophysiological study**. Blood FK506 concentration (x-axis) and amplitude of action potentials evoked in the hypothenar muscles (y-axis) of each animal 12 weeks after transplantation. The FK506 concentration correlated significantly with the amplitude of the muscle action potentials (correlation coefficient = 0.88, p = 0.002). (B) Blood FK506 concentration (x-axis) and motor nerve conduction velocity (y-axis) of each animal 12 weeks after transplantation. The FK506 concentration did not correlate significantly with the MNCV (correlation coefficient = 0.70, p = 0.054).

Twelve weeks after transplantation, all animals in the PA0.1 and the PA0.05 groups displayed evoked action potentials in the right hypothenar muscles, whereas no animal in the PA0.025 group displayed evoked action potentials in the right hypothenar muscles. The mean %muscle action amplitude was 100% (range 69.8-151.4%) in the PA0.1 group and 34.7% (range 9.7-60.6%) in the PA0.05 group. The mean %MNCV was 80.1% in the PA0.1 group and 61.4% in the PA0.05 group. The blood FK506 concentration and %muscle action potential amplitudes correlated significantly (correlation coefficient = 0.88, p = 0.002). The blood FK506 concentration and %MNCV did not correlate significantly (correlation coefficient = 0.70, p = 0.054) (Figure 2).

### Morphological study

**Figure 3:**
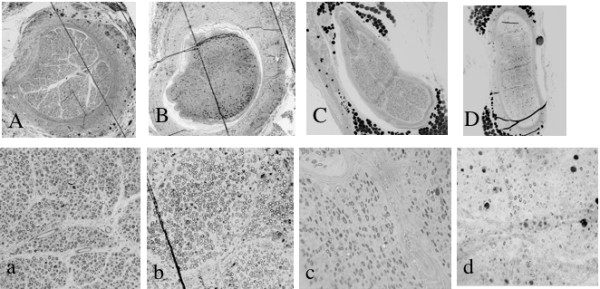
**Morphological study**. Light microscopic examination of transverse sections of the left ulnar nerve 1.5 cm distal to the distal neurorrhaphy of the nerve isograft (A, a) and allografts (B-D and b-d) 12 weeks after transplantation. (A-D) 100× magnification and a-d; 400× magnification. (A, a) Nerve isograft in the PL0.1 group; (B, b) nerve allografts in the PL0.1 group; (C, c) nerve allografts in the PL0.05 group; (D, d) nerve allografts in the PL0.025 group. (D, d) A very few myelinated axons were seen in the PL0.025 group.

**Figure 4:**
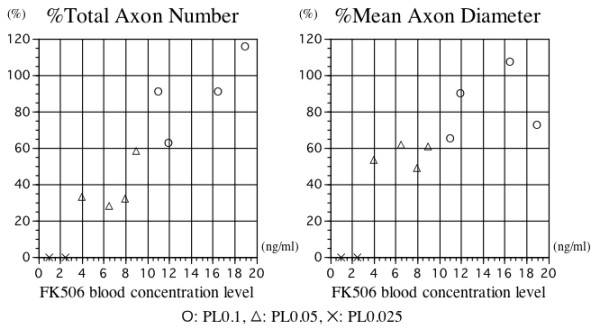
**Morphological study with blood FK 506 concentration**. Blood FK506 concentration (x-axis) and %-total axon number (y-axis) of each animal 12 weeks after transplantation. The FK506 concentration correlated significantly with the number of regenerated axons (correlation coefficient = 0.88, p = 0.002). The %-total axon number of each animal is expressed as the percentage of the total myelinated axon number in the right ulnar nerve divided by that in the left ulnar nerve. (B) Blood FK506 concentration (x-axis) and %-mean axon diameter (y-axis) of each animal 12 weeks after transplantation. The FK506 concentration did not correlate significantly with the mean axon diameter (correlation coefficient = 0.70, p = 0.054). The %-mean axon diameter of each animal is expressed as the percentage of the mean myelinated axon diameter in the right ulnar nerve (allogeneic nerve transplantation) divided by that in the left ulnar nerve (isogeneic nerve transplantation).

There were marked differences in the pattern of nerve regeneration between the groups (Figure 3). Myelinated axons were seen in all transverse sections taken about 1.5 cm distal to the distal neurorrhaphy of the right ulnar nerves in the PA0.1 and PA0.05 groups, whereas few myelinated axons were observed in the nerve allografts of the PA0.025 group. This finding suggests that the polyphenol treatment combined with the subcutaneous administration of FK506 at the low dose of 0.05 mg/kg every other day did not prevent immune rejection of the transplanted nerve segments. The mean %total axon numbers were 86.8% in the PA0.1 group, 35.0% in the PA0.05 group, and 0% in the PA0.025 group. The average %mean axon diameters were 80.2% in the PA0.1 group, 52.2% in the PA0.05 group, and 0% in the PA0.025 group. The blood FK506 concentration correlated significantly with the %total axon number (correlation coefficient = 0.91, p < 0.001). The FK506 concentration did not correlate significantly with the %mean axon diameters (correlation coefficient = 0.56, p = 0.153) (Figure 4).

### *Blood FK506 *concentration

The blood FK506 concentration differed markedly between dogs, even within the same group. The concentrations were 11-19 ng/ml (mean 14.6 ng/ml) in the PA0.1 group, 4-9 ng/ml (mean 6.9 ng/ml) in the PA0.05 group, and 1.0 and 2.5 ng/ml in the two dogs in the PA0.025 group.

### Genomic study

**Figure 5:**
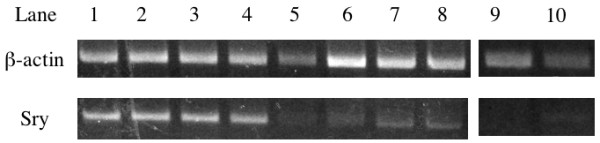
**Genomic study**. PCR specific to canine *Sry *and β-actin. (Lanes 1-4) PL0.1 group, (lanes 5-8) PL0.05 group, (lanes 9 and 10) PL0.025 group. (Upper row) β-actin-specific bands, (lower row) *Sry*-specific bands. β-actin-specific bands, but not *Sry*-specific bands, were seen in the specimens from the PL0.025 group.

Sufficient genomic DNA to perform semiquantitative PCR was extracted from all nerve allograft specimens in each group. The concentrations of the extracted DNA were 321.2-406.5 μg/ml in the PA0.1 group, 272.8-359.7 μg/ml in the PA0.05 group, and 81.8 μg/ml and 120.6 μg/ml in the PA0.025 group. Less DNA was extracted from nerve specimens in the PA0.025 group than in the PA0.1 and PA0.05 groups. PCR products specific to β-actin were detected in all samples in all groups. *Sry*-specific bands were detected in all samples in the PA0.1 and PA0.05 groups, but neither specimen demonstrated the *Sry*-specific band in the PA0.025 group (Figure 5). These results indicate that cells originating from the donor nerves remained in the nerve allografts in the PA0.1 and PA0.05 groups but that no cells of donor origin survived in the nerve allografts in the PA0.025 group.

## Discussion

The present study demonstrated that the extent of nerve regeneration of nerve allografts preserved in polyphenols for one month in association with the administration of 0.1 mg/kg/day of FK506 was more than 80% of that achieved with fresh nerve isografts. The %total myelinated axon number and %muscle action potential amplitude correlated strongly with the blood FK506 concentration in the recipient animals. PCR of the *Sry *region showed that cells originating in the allogeneic-transplanted nerve segments survived in the transplanted segments in dogs given 0.05 mg/kg/day of FK506.

FK506 (tacrolimus) is an immunosuppressant drug used widely in humans to prevent immune rejection of organ or tissue transplants [11-13]. The clinical literature shows that in humans, a dose of 10-20 ng/ml of FK506 is adequate to prevent rejection and that this dose is less than that associated with adverse effects following allogeneic organ or tissue transplantation [11-13]. We used a smaller dose in our study. Oike and Talpe reported that a blood FK506 concentration of 7-20 ng/ml led to successful allogeneic liver transplantation in miniature swine that were genetically semi-identical [19,20]. We observed nerve regeneration > 80% of that of the isogeneic nerve transplantation in the PA0.1 group, whose blood FK506 concentrations were > 10 ng/ml. Previous studies showed that nerve regeneration through fresh nerve allografts in association with a therapeutic dose of FK506 was similar to that of fresh nerve isografts [21,22]. Dogs transplanted with isogeneic sciatic nerve fascicles to the ulnar nerve might demonstrate almost the same level of nerve regeneration as those observed in the PA0.1 group.

High doses of FK506 have an anti-ischemic effect, and lower doses that maintain the blood FK506 concentration at 10-20 ng/ml do not show a tissue-protective effect against ischemia [23]. Polyphenols protected the nerve segments from ischemic damage when the transplanted nerve segments were preserved for one month. We wondered whether the polyphenol treatment of the transplanted nerve segments contributed to the suppression of immunological reactions following the allogeneic transplantation. Although the precise mechanisms of immunosuppression by polyphenols are not understood fully, several papers have suggested that epigallocatechin gallate (EGCG), the main component of polyphenols, has immunosuppressive actions [24-27]. Our previous study using a rat model demonstrated successful nerve regeneration in polyphenol-treated nerve segments transplanted between major histocompatibility-mismatched rats, although only 14% of the cells of donor origin remained in the nerve segments 12 weeks after the transplantation [10]. The degree of nerve regeneration using nerve allografts treated with polyphenols correlated significantly with the blood FK506 concentration in dogs in our present study. The immunosuppressive effects of polyphenols might not be strong enough to prevent immune system-induced tissue rejection following allogeneic transplantation in dogs, which have a more highly developed immune system compared with rodents.

In the present study, the donor sciatic nerve segment was divided into fascicles of < 1.5 mm, which were used for transplantation. Our previous studies demonstrated that immersion of a large-diameter nerve segment in the polyphenol solution for one month at 4°C was associated with the destruction of Schwann cells deeper than 0.7-0.8 mm below the perineural surface of the nerve segment. We suspected that the permeability of polyphenols into the peripheral nerve segments was < 0.7-0.8 mm from the perineural surface of nerves. The study revealed that the sciatic nerve segment should be divided into fascicles of < 1.4-1.6 mm before the polyphenol treatment [15]. It is known that FK506 has neuroprotective and neuroregenerative effects [28,29]. In the present study, we calculated the electrophysiological and morphological data of the nerve allografts as percentages relative to the nerve isografts in the contralateral forelimb to minimize the facilitative effect of FK506 on nerve regeneration.

Axons extended successfully through the nerve allografts that had been immersed in the polyphenol solution for one month when transplanted in association with a therapeutic dose of FK506 in a canine model. Although polyphenols have a strong anti-ischemic action, the ability to suppress immune rejection may not be strong enough to suppress the immunological rejection following allogeneic transplantation in animals such as the dog, which has a highly developed immune system.

## Conclusions

In contrast to our previous findings in a rodent model using 2-cm-long nerve allografts, immersion of nerve segments in the polyphenol solution alone did not prevent immune rejection following the allogeneic transplantation of the segments in dogs. Our data suggest that polyphenol immersion in association with a therapeutic dose of FK506 might lead to successful nerve regeneration in higher animals. The polyphenols protected the nerve allografts from ischemic damage but did not completely suppress immune rejection episodes induced by the allotransplantation against the grafts.

## Competing interests

The authors declare that they have no competing interests.

## Authors' contributions

RK designed the study and drafted the manuscript. KN, RK, RI performed experiment procedure, surgery. RK, RI, TY, SO, TN and SF performed evaluation of behavioral, biochemical and histopathological study. SD participated in the design of the study. All authors read and approved the final manuscript.
